# Characterization of anti-fentanyl antibodies as antagonists of ultra-toxic, clinically relevant or endogenous opioids

**DOI:** 10.1007/s00204-026-04331-0

**Published:** 2026-03-10

**Authors:** Franziska Endt, Dirk Steinritz, Niko Amend, Thomas Gudermann, Andreas Breit

**Affiliations:** 1 Walther Straub Institute of Pharmacology and Toxicology, Medical Faculty, LMU Munich, Goethestrasse 33, 80336 Munich, Germany; 2 Bundeswehr Institute of Pharmacology and Toxicology, Munich, Germany

**Keywords:** Therapeutic antibodies, Carfentanil, Fentanyl, Morphine, cAMP

## Abstract

The ultra-potent synthetic opioid carfentanil acts lethally by potently activating µ opioid receptors (µOR). Treatment based on competitive antagonists is of limited use due to the ultra-high affinity of the carfentanil/µOR complex. Thus, preventing formation of this complex by car neutralizing antibodies might be a promising alternative strategy. We tested nine antibodies raised against fentanyl (ab-fen) in receptor-ligand binding and receptor activation assays using µOR expressing HEK293 cells in the presence of fen or car. When high antibody concentrations (500 nM) were pre-incubated with opioids, seven ab-fen significantly inhibited fen binding to the µOR completely and four car binding up to 90 %. None of the tested antibodies affected remifentanil, morphine or endomorphine-1. Concentration-response curves revealed IC_50_-values of ab-fen between 25 and 74 nM against fentanyl and between 121 and 900 nM against carfentanil. Hill-slopes against fen were way above one (2.7–6.0), indicating extremely high positive cooperativity of ab-fen, which was not observed against carfentanil. Furthermore, low antibody concentrations (1.0 nM) enhanced fentanyl- and to a lesser extent carfentanil-induced µOR activation, indicating bi-functional actions of ab-fen. Moreover, when lethal carfentanil concentrations were first added to cells, ab-fen also disrupted the µOR/carfentanil complex with carfentanil still being present. Overall, ab-fen maybe able to stop and reverse carfentanil intoxications *in vivo* and their effects on opioid efficacy at low ab-fen concentrations suggest that they might be used in a new way to improve opioid-based pain therapy. Our findings might pave the way for future antibody development and refinement.

## Introduction

In accordance to the world health organization, ~ 600,000 deaths are attributed to drug use worldwide. Close to 80 % of these deaths are related to opioids, with around 25 % of those deaths caused by opioid overdose. Thus, up to 125,000 people die annually due to opioid intoxication. Over the last two decades ultra-potent synthetic opioids (UPSO) such as fentanyl (FEN) and carfentanil (CAR), in addition to morphine (MOR) and heroin, gained more and more relevance in this aspect (Cowles et al. 2017; Delcher et al. 2025, 2020; Gustafsson et al. 2024; Jaimet 2017; Zawilska et al. 2021). Furthermore, there is a significant threat of CAR being misused as a chemical weapon of war or terrorism (Riches et al. 2012; Shafer 2019).

Opioids act on µ opioid receptors (µOR), which are G protein-coupled receptors expressed in neurons of the CNS (Pathan and Williams 2012). Down-stream of µOR, opioids activate trimeric G proteins of the G_i/o_ family. G_i/o_α subunits inhibit activity of adenylyl cyclase and thus reduce cytosolic cAMP levels. G protein βγ-units released from G_i/o_α activate inwardly rectifying potassium channels and inhibit voltage-dependent calcium channels and thereby dampen neuronal activity (Montandon et al. 2016). Both ways have been proposed to contribute to respiratory depression and thus, to the toxic and deadly actions of opioids (Manzke et al. 2003; Pattinson 2008).

UPSO have been shown to exhibit ultra-high affinity, potency and efficacy to bind to and activate µOR in various cell models (Costa et al. 1992; Endt et al. 2025; Faouzi et al. 2023; Feasel et al. 2024; Hsu et al. 2019; Titeler et al. 1989). These *in vitro* pharmacodynamics lead to receptor over stimulation and translate into ultra-low lethal *in vivo* doses. FEN has been reported to be ~100-times and CAR ~10,000 times more lethal compared to MOR (George et al. 2010; Langston et al. 2020; Lust et al. 2011). Thus, the risk of respiratory depression after FEN or CAR intoxication is extremely high at even low doses making UPSO deadly drugs (Van Bever et al. 1974).

The µOR antagonists naloxone and nalmefene bind to the receptor with affinities in the nanomolar range and thus sufficiently displace opioids from the µOR (Endt et al. 2025; Feasel et al. 2024). Consequently, both antagonists are clinically used antidotes against opioid intoxication in the case of MOR and heroin (Bell and Strang 2020). However, in the case of FEN and particular CAR, competitive antagonists failed as antidotes *in vivo* and *in vitro* due to the high affinity and potency of the CAR/µOR complex (Leen and Juurlink 2019; Zawilska et al. 2021). In addition, data from others and our group have shown that CAR is resistant to µOR antagonist on the cellular level, because it acts as a biased agonist that stabilizes µOR conformations with very low affinity for antagonists (Alhosan et al. 2025; Endt et al. 2025; Feasel et al. 2024). In line with these data, µOR antagonist were less potent at reversing the effects of CAR compared to heroin on ventilatory depression in rats (Flynn and France 2025). Thus, there is an urgent need for alternative approaches against UPSO intoxications that prevent the formation of the high-affinity CAR/µOR complex and/or disrupts it.

Over the past years, antibodies gained more and more recognition as therapeutic tools in medicine in general and as antidotes against FEN intoxication in particular (Sharma et al. 2023). Several ab-FEN have been shown to prevent FEN toxicity in rats (Khaimraj et al. 2024; Ribaudo et al. 2025). In order to evoke toxic effects, inhaled or injected UPSO have to enter the CNS via the blood-brain-barrier (BBB). Because of their specific binding to the UPSO and their inability to cross the BBB, antibodies prevented crossing of the BBB by FEN and thus decreased its brain and increased its serum concentration until it was metabolized or excreted. Of note, recent studies have shown, that monoclonal antibodies raised against FEN could also shield against the *in vivo* effects of CAR in mice (Eubanks et al. 2021; Galbo-Thomma et al. 2025; Urban et al. 2024). Thus, antibodies against UPSO revealed their potential as antidotes against FEN and maybe even CAR *in vivo*. However, no data about the pharmacodynamics of such antibodies in terms of affinity, selectivity, efficacy, cooperativity or kinetics are available when these antibodies interact with distinct opioids in the presence of the receptor in living cells. Such data could help to discover new antibodies and to better understand their modes of actions in particular when antibodies could reach the CNS after appropriate modifications (Platt et al. 2017; Zhao et al. 2022b).

In this study, we analyzed nine commercially available antibodies originally raised against FEN (ab-FEN) for their ability to inhibit the formation of µOR complexes with various opioids in membrane fractions or living cells. Further, we analyzed the consequences of ab-FEN binding to opioids on µOR activation. We found that seven ab-FEN neutralized FEN and four CAR, with one being specific against CAR. None of the ab-FEN interfered with clinically relevant opioids (MOR, REMI) or the endogenous opioid ENDO. We found affinities in the nanomolar range, positive cooperativity and bi-functional effects, in particular against FEN. Further, we can show that ab-FEN are not only able to prevent the formation of the CAR/µOR complex but also to disrupt it even in the presence of lethal CAR concentrations. Overall, our data highlight the potential of antibodies as antidotes against UPSO and reveal first information about their pharmacodynamics, which might be helpful for their future development and refinement.

## Materials and methods

### Chemicals and antibodies

MOR (M-005), FEN (F-013), CAR (C-163), REM (R-024), ENDO (SCP-0132), forskolin (FSK) (F3917) and 3-isobutyl-1-methylxanthin (IBMX) (I5879) were purchased from SigmaAldrich (St Louis, USA). [N-allyl-2,3,-^3^H]-naloxone (NET-719250UC) was from PerkinElmer (Boston, USA). Anti-fentanyl antibodies (ab-FEN) were either from Medix Biochemica (Espoo, Finnland), Biozol (Hamburg, Germany) or Invitrogen (Waltham, Massachusetts, USA) and are listed in Table 1.

**Table 1:** List of antibodies used in the present study

Antibody	Provider	Purchase order number	Immunogen	Type
Ab-FEN-1	Medix Biochemica	HM1132-1	Fentanyl-KLH	Monoclonal
Ab-FEN-2	Medix Biochemica	HM004	Fentanyl-KLH	Monoclonal
Ab-FEN-3	Medix Biochemica	HM628-1	Fentanyl-KLH	Monoclonal
Ab-FEN-4	Medix Biochemica	HM1133-1	Fentanyl-KLH	Monoclonal
Ab-FEN-5	Medix Biochemica	100974	Fentanyl-BSA	Monoclonal
Ab-FEN-6	Medix Biochemica	100998	Fentanyl-BSA	Monoclonal
Ab-FEN-7	Biozol	ECB-P01-99-54R-IF	Fentanyl-BgG	Polyclonal
Ab-FEN-8	Medix Biochemica	P01-9953R-IF	Fentanyl-BgG	Polyclonal
Ab-FEN-9	Invitrogen	PA175148	Fentanyl-BSA	polyclonal

### Cell culture

HEK293 cells were cultured in DMEM + GlutaMAX^TM^ containing 10 % fetal bovine serum and 100 U/ml penicillin and streptomycin from Gibco. HEK293-µOR cells have previously been published (Endt et al. 2025).

### Radioligand binding assay

**Fig. 1:**
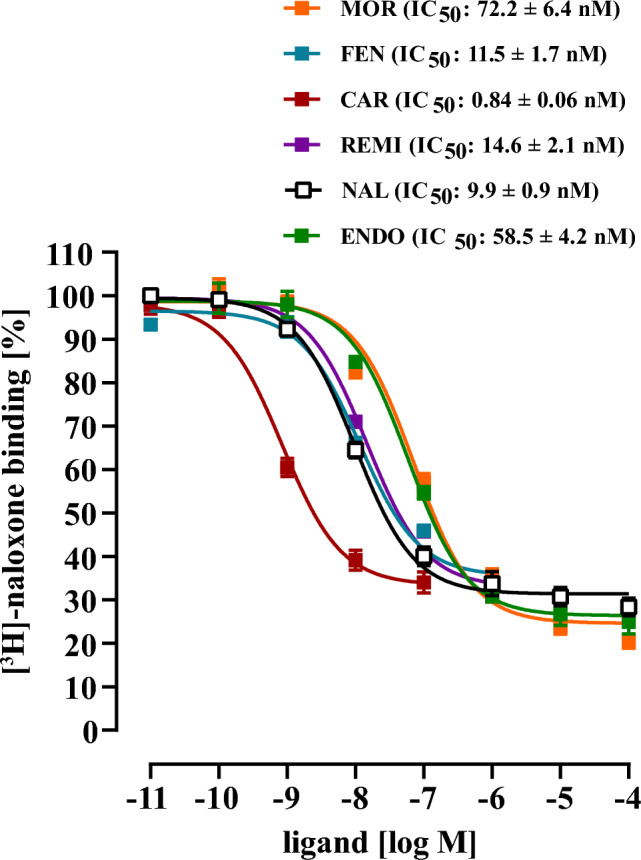
Competition binding with [^3^H]-naloxone and total membrane fractions of HEK293-µOR cells. 5 nM [^3^H]-naloxone competed with increasing opioid concentrations as indicated. Corresponding IC_50_-values are shown in parenthesis. Data are show as percentage of total [^3^H]-naloxone binding and results from 4 independent experiments (N = 4) performed in triplicates are presented as the mean ± SEM

**Fig. 2:**
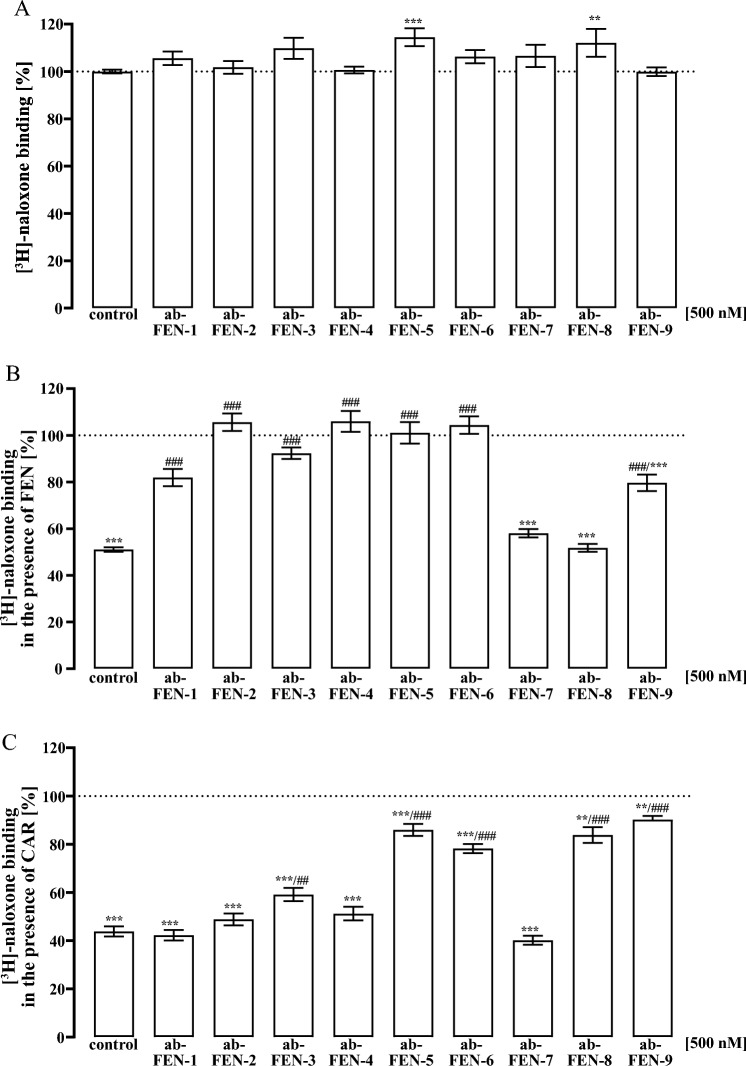
Competition binding with [^3^H]-naloxone (5 nM) and total membrane fractions of HEK293-µOR cells. In **A** total [^3^H]-naloxone was detected after pre-incubating tracer for 30 min at 37 °C with 500 nM of the indicated ab-FEN. In **B** [^3^H]-naloxone competed with FEN (150 nM) and in **C** with CAR (10 nM) pre-incubated with the indicated ab-FEN (30 min, 37 °C). Data are show as percentage of [^3^H]-naloxone binding and results from 5 independent experiments (N = 5) performed in triplicates are presented as the mean ± SEM. Statistical differences were calculated using one‐way ANOVA followed by Tukey´s post-test. Asterisks indicate significant differences to [^3^H]-naloxone alone, hash signs to [^3^H]-naloxone and opioid

**Fig. 3:**
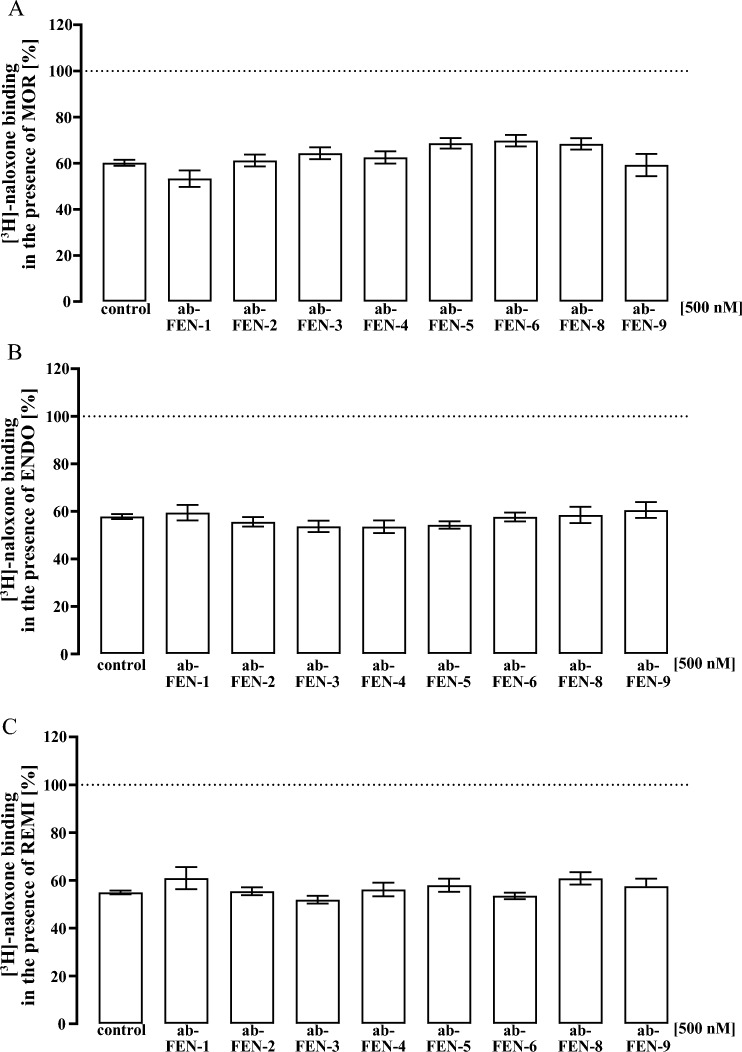
Competition binding with [^3^H]-naloxone (5 nM) and total membrane fractions of HEK293-µOR cells. In **A** [^3^H]-naloxone competed with MOR (200 nM), in **B** with ENDO (2 µM) and in **C** with REMI (500 nM) pre-incubated with the indicated ab-FEN (30 min, 37 °C). Data are shown as percentage of [^3^H]-naloxone binding and results from 5 independent experiments (N = 5) performed in triplicates are presented as the mean ± SEM

For data provided in Figs. 1, 2 and 3, total membrane fractions were first prepared as described previously and aliquots stored as − 80 °C (Breit et al. 2004). 20 µg of membranes were incubated with 5 nM of [^3^H]-naloxone in DMEM. Specific [^3^H]-naloxone was determined as the inhibition of total [^3^H]-naloxone by increasing concentrations of NAL, FEN, CAR, REMI, MOR or ENDO (Fig. 1), or with FEN (150 nM) or CAR (10 nM) in Fig. 2 and MOR (200 nM), ENDO (2 µM) or REMI (500 nM) in the presence or absence of the indicated ab-FEN (500 nM) in Fig. 3. Opioids were first incubated with the antibodies for 30 min at 37 °C in DMEM, then mixed with the radiolabeled tracer (5 nM of [^3^H]-naloxone) and then the reaction was started by adding the cell membranes. Samples were incubated at 37 °C for 30 min and the reaction stopped by rapid filtration over Whatman GF/C glass-fibers filters (VWR, Radnor, USA) using a cell harvester from Brandel (Gendex, Glasgow, UK). Remaining radioactivity was measured by scintillation counting using a WinSpectral1414 (PerkinElmer, Boston, USA). For data shown in Figs. 6, 7 and 8, ~ 60,000 cells were seeded on poly-L-lysin coated 48-well plates and, serum-starved after 24 h for another 24 h. For data shown in Fig. 6, medium was replaced by 100 µl of DMEM containing 5 nM of [^3^H]-naloxone either alone or together with increasing concentration of FEN or CAR. After 30 min at 37 °C, cells were washed twice with 300 µl of PBS and lysed with 100 µl of 1 % SDS and 1 N NaOH. Remaining radioactivity in the lysates was measured by scintillation counting using a WinSpectral1414 (PerkinElmer, Boston, USA). For data provided in Fig. 7, medium was replaced with DMEM containing 5 nM of [^3^H]-naloxone, 150 nM FEN or 10 nM CAR alone or together with increasing concentrations of the corresponding antibody. These samples were incubated for 30 min at 37 °C and then added to cells for another 60 min at RT. Washing and scintillation counting performed as described above. For data shown in Fig. 8, [^3^H]-naloxone, FEN or CAR was first added to the cells for 30 min at 37 °C and then the corresponding antibody (500 nM) was added for the indicated period of time at RT.

### cAMP accumulation

**Fig. 4:**
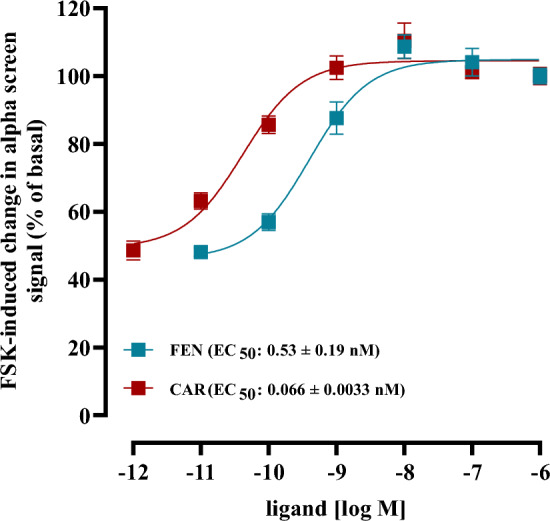
Detection of cAMP by alpha-screen in HEK293-µOR cells. Cells were pre-incubated with IBMX (500 µM, 5 min) stimulated with FSK (1 µM) alone or along with increasing concentrations of either FEN or CAR. Corresponding IC_50_-values are shown in parenthesis. Data are shown as percentage of FSK-induced reduction of the cAMP-dependent alpha-screen signal and results from 4 independent experiments (N = 4) performed in triplicates are presented as the mean ± SEM

**Fig. 5:**
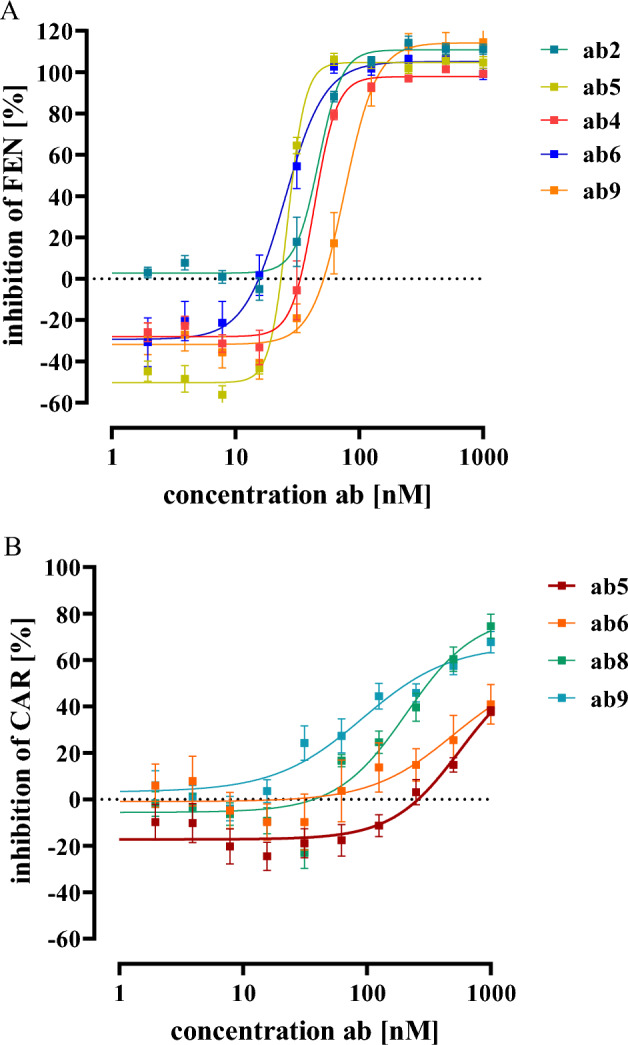
Detection of cAMP by alpha-screen in HEK293-µOR cells. Cells were pre-incubated with IBMX (500 µM, 5 min) stimulated with FSK (1 µM) alone or along with either FEN (15 nM) **A** or CAR (1 nM) **B** pre-incubated (30 min, 37 °C) with increasing concentrations of various ab-FEN. Data are shown as percentage of opioid-induced reduction of the FSK signal and results from 5 independent experiments (N = 5) performed in triplicates are presented as the mean ± SEM

Alpha-screen cAMP detection kit (6760635D) from Revvity was used to detect cytosolic cAMP. ~ 30,000 cells were seeded on poly-L-lysin coated 96-well plates and, serum-starved after 24 h for another 24 h. Cells were then incubated with 50 µl IBMX (500 µM) in DMEM for 5 min and stimulated with 50 µl DMEM containing FSK (1µM) and IBMX, opioids or antagonists 2-fold concentrated (Fig. 4). In Fig. 5 FEN (15 nM) or CAR (1 nM) was first incubated with increasing ab-FEN concentrations for 30 min at 37 °C. After 20 min at 37 °C, stimulation was stopped by removal of the medium and addition of 40 µl lysis puffer containing acceptor beads (12.5 µg/ml) and biotinylated cAMP (0.625 pmole). After 90 min, 10 µl lysis puffer with donor beads (12.5 µg/ml) was added and incubated for additional 30–60 min. Finally, acceptor bead emission (570 ± 100 nm) was detected after excitation of the donor bead (680 ± 40 nm) using a ClarioStar from BMG (Offenburg, Germany).

### Quantification and statistical analysis

Values represent the mean ± SEM of 3–6 independent experiments. Statistical analysis was performed using one- or two-sample student’s t-test, one‐way or two-way ANOVA followed by Tukey´s or Dunnett´s post-test using the GraphPad prism software 9.1 (RRID:SCR_002798). Shapiro-Wilk tests were performed in order to ensure normal distribution of the data sets. One symbol indicates a *p*‐value of ≤ 0.05, two of ≤ 0.01 and three of ≤ 0.001.

## Results

### Anti-FEN antibodies inhibit FEN and CAR but not REMI, MOR or ENDO binding to the µOR

Standard testing of antibodies against bioactive small molecules includes biochemical assays such as ELISA techniques, which measure antibody binding to the corresponding antigen *ex vivo* and thus isolated from their biological actions in the absence of the cognate target. We aimed at dissecting the pharmacodynamics of various commercially available antibodies raised against FEN (ab-FEN) with cells expressing the µOR. Firstly, defining affinities of ab-FEN under these rather biological settings might better mirror the *in vivo* situation, and secondly, no data about the effects of ab-FEN on UPSO in the presence of the µOR are available. In a first step, we established an assay that monitors ab-FEN actions on opioid receptor binding, which represents the first and initial step in their clinical and toxic actions. To this end, we used recently established HEK293-µOR cells and performed competition binding experiments with [^3^H]-naloxone and total membrane fractions. As shown in Fig. 1, all tested opioids showed monophasic competition curves against [^3^H]-naloxone with a rank order of affinity CAR > FEN = REMI = NAL > MOR = ENDO and the indicated IC_50_-values between 0.8 and 72 nM. These data are in good agreement with previously published results (Costa et al. 1992; Endt et al. 2025; Faouzi et al. 2023; Feasel et al. 2024; Hsu et al. 2019; Titeler et al. 1989). Thus, we tested next whether ab-FEN could interfere with opioids and thereby prevent the formation of the opioid/receptor complex. We tested a rather high ab-FEN concentration (500 nM) of the nine antibodies listed in Table 1. As a control, we first tested whether ab-FEN would interfere with [^3^H]-naloxone binding to the µOR. We incubated the corresponding antibodies for 30 min with [^3^H]-naloxone at 37 °C and tested then [^3^H]-naloxone binding compared to the control without antibody. As indicated in Fig. 2A, none of tested antibody reduced the formation of [^3^H]-naloxone/µOR complexes, Indicating no significant interactions between the antagonist and ab-FEN. Next, we incubated ab-FEN first with FEN (150 nM) for 30 min at 37 °C, added the complexes to cell membranes with [^3^H]-naloxone and monitored then remaining tracer occupancy by the receptor. Compared to the FEN control, which reduced [^3^H]-naloxone occupancy to 51.1 ± 1.0 %, all ab-FEN, besides ab-FEN-7 and -8, restored [^3^H]-naloxone occupancy almost completely (Fig. 2B, Table 2), indicative for functional interactions between these antibodies and the opioid that block formation of µOR ligand complexes. Encouraged by these findings, we tested ab-FEN under the same setting against CAR, but reduced the opioid concentration to 10 nM, due to its ~ 15-times higher affinity compared to FEN shown in Fig. 1. Interestingly, ab-FEN-5, -6, -8 and -9 restored [^3^H]-naloxone occupancy to µOR in the presence of CAR up to 90 % (Fig. 2C, Table 2). Hence, antibodies originally raised against FEN, exhibit sufficient cross-activity towards CAR, so that formation of the high-affinity complex with µOR was significantly inhibited. Of note, ab-FEN-8, which had no effect on FEN, significantly restored [^3^H]-naloxone occupancy in the presence of CAR from 43.8 ± 2.1 to 83.8 ± 3.3 %, and thus showed selectivity towards CAR. Next, we tested ab-FEN that neutralized either CAR or FEN for their ability to antagonize the clinically relevant opioids REM and MOR or the endogenous opioid ENDO. As shown in Fig. 3 and summarized in Table 2, none on the ab-FEN tested had any effect on either opioid, indicating selectivity of the antibodies towards FEN and CAR.

**Table 2:** Effects of various antibodies (500 nM) on opioid binding to membrane preparations of HEK293-µOR cells (% of remaining [^3^H]-naloxone binding to the µOR)

	FEN 150 nM	CAR 10 nM	MOR 200 nM	ENDO 2 µM	500 nM
Control	51.1 ± 1.0	43.8 ± 2.1	60.2 ± 1.3	57.8 ± 1.0	54.9 ± 0.8
Ab-FEN-1	81.9 ± 3.6***	42.3 ± 2.2	63.3 ± 3.5	59.4 ± 3.2	60.1 ± 4.7
Ab-FEN-2	105.6 ± 3.7***	48.8 ± 2.4	61.2 ± 2.6	55.6 ± 2.0	55.4 ± 1.7
Ab-FEN-3	92.3 ± 2.4***	59.1 ± 2.7	64.3 ± 2.6	53.7 ± 2.4	51.9 ± 1.6
Ab-FEN-4	106.0 ± 4.5***	51.2 ± 2.8	62.5 ± 2.6	53.5 ± 2.7	56.2 ± 2.9
Ab-FEN-5	101.1 ± 4.6***	86.0 ± 2.5***	68.6 ± 2.3	54.3± 1.5	57.9 ± 2.7
Ab-FEN-6	104.3 ± 3.7***	78.2 ± 1.9***	69.8 ± 2.5	57.6 ± 1.9	53.3 ± 1.4
Ab-FEN-7	58.0 ± 1.7	40.2 ± 1.9	nd	nd	nd
Ab-FEN-8	51.8 ± 1.7	83.8 ± 3.3***	68.4 ± 2.5	58.5 ± 3.4	60.8 ± 2.6
Ab-FEN-9	79.7 ± 3.5***	90.2 ± 1.5***	59.2 ± 4.8	60.5 ± 3.3	57.5 ± 3.2

Statistical differences were calculated using one‐way ANOVA followed by Tukey´s post-test. Asterisks (***) indicate significant differences to the control

### Anti-FEN antibodies block FEN- and CAR-induced µOR activation in the nanomolar range, with high co-operativity and in a bifunctional manner

After establishing a cell-based assay that allows sensitive and robust detection of ab-FEN interactions with FEN and CAR, we analyzed effects of ab-FEN on opioid-induced µOR activation in living cells. Since µOR are Gi/o-coupled receptors, determination of cytosolic cAMP levels is a well-established technique to monitor µOR activity. Alpha-screen cAMP assays detect cytosolic cAMP levels by a decreased signal in the presence of increased cAMP. Consequently, the direct adenylyl cyclase activator Forskolin (FSK) decreased the cAMP-dependent alpha-screen signal and increasing FEN or CAR concentration restored it back to basal, indicative for opioid-induced G_i/o_ activation via µOR (Fig. 4). IC_50_-values were 0.53 ± 0.19 nM for FEN and 0.07 ± 0.003 nM for CAR, which is in good agreement with previous data (Costa et al. 1992; Endt et al. 2025; Faouzi et al. 2023; Feasel et al. 2024; Hsu et al. 2019; Titeler et al. 1989). Thus, we next induced µOR activation with either 15 nM of FEN or 1 nM of CAR after pre-incubation (30 min at 37 °C) with various ab-FEN concentrations (0.1–1 µM). Thereby, we were firstly able to monitor whether ab-FEN are indeed able to prevent opioid-induced receptor activation in living cells and secondly to calculate the corresponding pharmacodynamics such as IC_50_-value, efficacy and cooperativity. Based on the data shown in Fig. 2, we chose to test ab-FEN-2, -4, -5, -6 and -9 against FEN and ab-FEN-5, -6, -8 and -9 against CAR. For FEN, we found IC_50_-values between 25 and 74 nM, with ab-FEN-5 and -6 being the most affine antibodies (Fig. 5, Table 3). Efficacy of all ab-FEN was also very high, because FEN-induced µOR activation was completely blocked. Furthermore, we observed two so far unrecognized characteristics of the interactions between antibodies and opioids: (1) hill-slopes were 2.7–6.0, indicating strong positive cooperativity and (2), at low antibody concentrations (1.0 nM), FEN inhibited FSK-induced cAMP accumulation even stronger with ab-FEN compared to the control without any antibody, indicating that at low ab-FEN concentrations the opioid activated the µOR with even higher efficacy. For CAR, IC_50_-values between 120 and 900 nM were observed, with ab-FEN-9 having the highest affinity. Ab-FEN-8, which showed no effects on FEN in the binding assay, exhibited an IC_50_-value of 224 ± 74 nM to CAR, indicating selectivity of this antibody with rather high affinity. This, antibody blocked 79.0 ± 6.8 % of CAR-induced µOR activation and thus, had the highest efficacy. Of note, none of the ab-FEN increased CAR´s efficacy at low concentrations and hill-slopes were with the exception of ab-FEN-9 ~ 1.0, suggesting no cooperativity of the antibodies against CAR.

**Table 3:** Effects of various antibodies (0.1–1000 nM) on opioid-induced cAMP inhibition in living HEK293-µOR cells

	Fentanyl (15 nM)				Carfentanil (1 nM)			
	IC_50_ [nM]	Hill slope	effect at 1.0 nM [%]	effect at 1.0 µM [%]	IC_50_ [nM]	Hill slope	effect at 1.0 µM [%]	effect at 1.0 µM [%]
Ab-FEN-2	45.5 ± 8.8	4.6 ± 0.6	2.7 ± 0.8	111 ± 7.4	nd	nd	nd	nd
Ab-FEN-4	42.6 ± 7.3	4.8 ± 0.9	− 28 ± 2.6	98 ± 3.6	nd	nd	nd	nd
Ab-FEN-5	26.9 ± 1.5	6.0 ± 1.1	− 50 ± 5.4	105 ± 4.4	872 ± 380	1.6 ± 0.4	− 17 ± 3.2	59 ± 5.6
Ab-FEN-6	25.7 ± 3.7	2.7 ± 0.6	− 29 ± 3.2	105 ± 5.2	904 ± 473	1.3 ± 0.7	− 1 ± 0.6	56 ± 3.8
Ab-FEN-8	nd	nd	nd	nd	224 ± 74	1.5 ± 0.3	− 6 ± 0.6	79 ± 6.8
Ab-FEN-9	73.5 ± 19	3.3 ± 1.2	− 31 ± 4.1	114 ± 8.6	121 ± 23	3 ± 0.9	3 ± 2.2	67 ± 4.4

nd, not determined

**Fig. 6:**
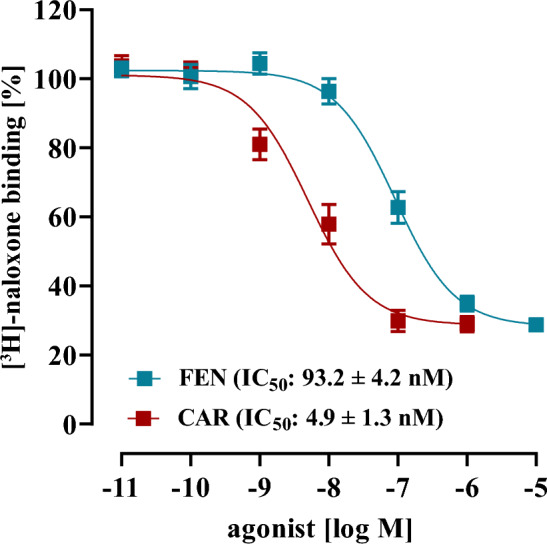
Competition binding with [^3^H]-naloxone and intact HEK293-µOR cells. 5 nM [^3^H]-naloxone competed with increasing opioid concentrations as indicated. Corresponding IC_50_-values are shown in parenthesis. Data are shown as percentage of total [^3^H]-naloxone binding and results from 4 independent experiments (N = 4) performed in triplicates are presented as the mean ± SEM

**Fig. 7:**
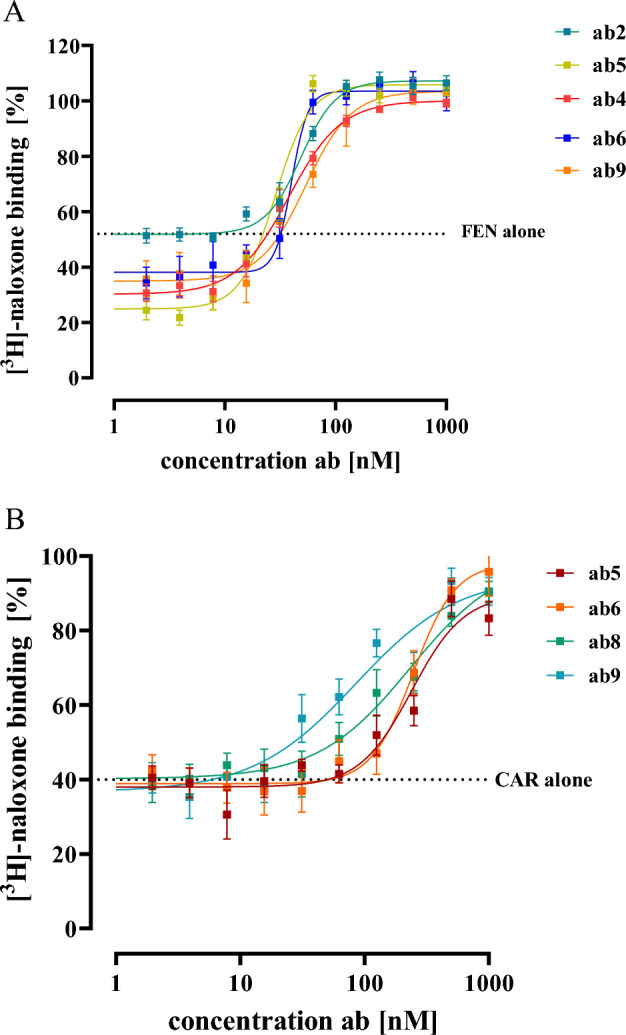
Competition binding with [^3^H]-naloxone and intact HEK293-µOR cells. 5 nM [^3^H]-naloxone competed with FEN (150 nM) in **A** or with CAR (10 nM) in **B** pre-incubated (30 min, 37 °C) with the indicated ab-FEN concentrations for 60 min at RT. Data are shown as percentage of total [^3^H]-naloxone binding and results from 4 independent experiments (N = 4) performed in triplicates are presented as the mean ± SEM

In order to correlate the pharmacodynamics of ab-FEN on FEN/CAR-induced receptor activation with receptor binding, we next aimed at performing [^3^H]-naloxone competition binding assay similar to those shown in Figs. 2 and 3 with intact cells and decreasing ab-FEN concentrations. First, we determined IC_50_-values of CAR and FEN with intact cells (Fig. 6). We observed an IC_50_-value of 93.2 ± 4.2 nM for FEN and of 4.9 ± 1.3 nM for CAR. Compared to our data obtained with total membrane fractions shown in Fig. 1, both ligands exhibited slightly reduced affinities in intact cells. Thus, we detected now [^3^H]-naloxone occupancy to the µOR in the presence of 150 nM FEN or 10 nM CAR, respectively, after pre-incubation with various ab-FEN concentrations (Fig. 7). IC_50_-values of ab-FEN in inhibiting FEN/µOR complexes in intact HEK293 cells were in the range of 75 to 132 nM, and thus ~ 2-fold higher compared to their effects on opioid-induced cAMP inhibition, shown before (Fig. 5 and Table 3). Hill-slopes were between 2 and 6, and low ab-FEN concentrations increased the ability of FEN to displace [^3^H]-naloxone (Fig. 7, Table 4). Thus, positive cooperativity as well as opioid activity enhancing effects of ab-FEN occurred on the level of ligand receptor binding. When formation of CAR/µOR complexes were monitored, affinity of ab-FEN was also slightly worse in the binding assay compared to activation assay, however, ab-FEN-9 showed low affinity binding to CAR with an IC_50_-value of 179 ± 23 nM.

**Table 4:** Effects of various antibodies (0.1–1000 nM) on opioid binding to living HEK293-µOR cells

	Fentanyl (150 nM)				Carfentanil (10 nM)			
	IC_50_ [nM]	Hill slope	effect at 1.0 nM [%]	effect at 1.0 µM [%]	IC_50_ [nM]	Hill slope	effect at 1.0 nM [%]	effect at 1.0 µM [%]
Ab-FEN-2	90.3 ± 7.7	2.6 ± 0.9	0.1 ± 2.9	107 ± 3.7	nd	nd	nd	nd
Ab-FEN-4	75.4 ± 8.6	1.7 ± 0.5	22 ± 3.5	100 ± 7.5	nd	nd	nd	nd
Ab-FEN-5	74.9 ± 5.9	3.5 ± 0.8	28 ± 4.1	105 ± 6.9	< 1000	1.1 ± 0.7	0.1 ±2.9	89.8 ± 4.8
Ab-FEN-6	82.9± 6.1	5.6 ± 1.4	14 ± 3.8	104 ± 8.2	555 ± 82	2.2 ± 0.7	0.4 ± 0.9	105 ± 7.2
Ab-FEN-8	nd	nd	nd	nd	594 ± 41	1.1 ± 0.2	0.1 ± 1.9	107 ± 4.7
Ab-FEN-9	132 ± 8.6	3.1 ± 0.7	20 ± 3.4	103 ± 4.8	179 ± 21	1.0 ± 0.3	10.2 ± 2.3	96.7 ± 8.6

nd, not determined

### Anti-FEN antibodies disrupt preformed opioid-µOR complexes

**Fig. 8:**
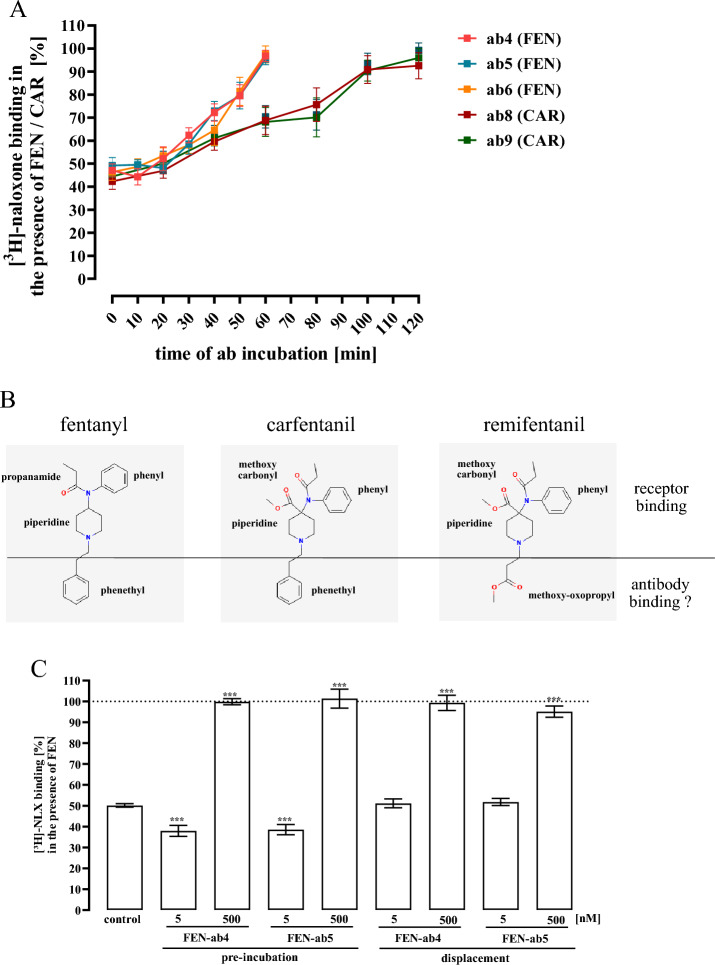
Competition binding with [^3^H]-naloxone and intact HEK293-µOR cells. In **A** 5 nM [^3^H]-naloxone competed with FEN (150 nM) or with CAR (10 nM) for 30 min at RT with intact cells. Afterwards cells were incubated with ab-FEN (500 nM) for the indicated periods of time. Data are show as percentage of total [^3^H]-naloxone binding and results from 5 independent experiments (N = 5) performed in triplicates are presented as the mean ± SEM. In **B**, structure of FEN, CAR and REMI provided by pubchem are presented. In **C**, intact HEK293-µOR cells were incubated with [^3^H]-naloxone (5 nM) either first with FEN (150 nM) and then with 5 or 500 nM ab-FEN (displacement) or FEN and ab-FEN were first pre-incubated. Data are shown as percentage of total [^3^H]-naloxone binding and results from 4 independent experiments (N = 4) performed in triplicates are presented as the mean ± SEM. Statistical differences were calculated using one‐way ANOVA followed by Tukey´s post-test. Asterisks indicate significant differences to the control

So far, we provide new data sets indicating that ab-FEN are able to prevent µOR complex formation with FEN or CAR when incubated with the opioid before it binds to the receptor, which would hinder crossing of the BBB by the UPSO *in vivo*. From a therapeutic standpoint, such a scenario would rather be described as pretreatment or prophylactic (vaccination-like) strategy. Recent advanced research established approaches that enable antibodies to cross the BBB and thus to act in the CNS (Zhao et al. 2022b). In order to test whether ab-FEN could disrupt preformed opioid-µOR complexes after entering the CNS and thus potentially be used after an opioid intoxication, we now added first [^3^H]-naloxone with either FEN or CAR to intact HEK293-µOR cells (30 min at 37 °C) and then ab-FEN for different time points. Ab-FEN-4, -5 or -6 restored [^3^H]-naloxone occupancy completely, and thus displaced FEN from µOR, within 60 min (Fig. 8A). Ab-FEN-8 and -9 acted similar on the CAR-µOR complex but restored maximal [^3^H]-naloxone occupancy only after 120 min, most likely due to a slower dissociation kinetic of the CAR-µOR complex (Fig. 8A). However, these data suggest that, ab-FEN are not only able to prevent formation of ultra-highly toxic CAR/OR complexes, but also to break them up in the presence of the UPSO in a therapeutic relevant time window.

The structure of FEN consists of four major functional groups: propanamide, phenyl, piperidine and phenethyl (Fig. 8B). Ab-FEN potentially bind to one or multiple areas of FEN. It has been proposed that the phenethyl group is less important for FEN´s interactions with the µOR (Vo et al. 2021). In line with these data, within the REMI molecule phenethyl is replaced by methoxy-oxopropyl without losing affinity (Figs. 1 and 8). Interestingly, CAR still contains the phenethyl group (Fig. 8). Because ab-FEN bind CAR but not REMI, one might assume that ab-FEN bind to FEN and CAR via the phenethyl group and thus in areas that are different from those interacting with µOR. In such a scenario, FEN could bind simultaneously to the µOR and ab-FEN. When FEN is pre-incubated with low ab-FEN concentrations, the ab-FEN/FEN complex could somehow increase affinity of FEN to the µOR. When the FEN-µOR complex is formed first, the addition of ab-FEN might be without such an effect. To shed light on this hypothesis, we tested ab-FEN-4 and -5 with low (5 nM) and high (500 nM) concentrations either pre-incubated with FEN or added to cells after the FEN-µOR complexes were preformed (displacement). As shown in Fig. 8C, 500 nM of both ab-FEN restored [^3^H]-naloxone occupancy under both settings completely. In contrast, 5 nM of ab-FEN decreased [^3^H]-naloxone binding to the µOR in the presence of FEN even further only when the ab-FEN/FEN complex was preformed and not when FEN was already bound to the receptor. Suggesting that the bi-functional actions of ab-FEN depend on the sequence of interactions between FEN, ab-FEN and the receptor, possibly due to distinct interfaces of FEN to interact with both binding partners.

## Discussion

The fentanyl crisis urgently demands the development of new therapeutic strategies against intoxication with UPSO. Because established competitive antagonist-based therapy fails due to the ultra-affinity binding of UPSO to the µOR, neutralizing UPSO with antibodies appears as an alternative strategy (Alhosan et al. 2025; Endt et al. 2025; Feasel et al. 2024; Flynn and France 2025; Leen and Juurlink 2019; Zawilska et al. 2021). Therapeutic antibodies against UPSO intoxication act by binding to the opioid in the serum and thereby hinder crossing of the BBB by the opioid. Such antibodies are tested and refined *ex vitro* or in the living animal. No data about the pharmacodynamics of such antibodies with living cells expressing the µOR are available. Thus, we developed cell-based assays that allow for testing the effects of antibodies on receptor binding and activation by various opioids. We found selective, high-affinity binding of ab-FEN to FEN and CAR and revealed so far unappreciated modes of ab-FEN actions, such as positive cooperativity and bi-functional actions.

First we monitored efficacy of nine commercially available ab-FEN at a rather high concentration in blocking opioid binding to the µOR in total membrane fractions. We found that seven ab-FEN blocked FEN completely and four CAR up to 90 %. MOR, ENDO and REMI were not affected. The effects of an antibody on opioid receptor binding depends on its affinity to the opioid and the opioid concentration tested. We did not used equal opioid concentrations but affinity adjusted concentrations (10 nM for CAR 200 nM MOR, respectively). Thus, our data do not necessarily indicate different affinities of ab-FEN to various opioids. Our goal was not to determine the real affinity of ab-FEN to opioids but to detect their effects on toxic FEN or CAR concentrations and clinically relevant MOR concentrations. 10 nM CAR correspond to ~ 5 ng/ml serum concentration. Blood CAR concentrations detected post mortem in individuals deceased from CAR overdoses were between 0.2 and 4 ng/ml and thus below the CAR levels blocked by ab-FEN tested herein (Elliott and Hernandez Lopez 2018; Fomin et al. 2018; Shanks and Behonick 2017; Swanson et al. 2017; Wallage et al. 2022). The same ab-FEN concentration did block clinically relevant MOR serum concentrations of 0.2 µM MOR, which are well below the serum concentrations of MOR overdose victims (Meissner et al. 2002). Thus, ab-FEN could potentially block fatal CAR concentration without affecting clinically relevant MOR doses. This, could be of high interest in scenarios of expected intoxications or even threatened attacks with CAR. CAR neutralizing antibodies could be applied in advance as a prevention and a morphine-based pain therapy would still be effective if necessary.

Using living cells, we also determined IC_50_-values of ab-FEN in blocking CAR or FEN receptor binding and activation. In the activation assay, we obtained IC_50_-values of ab-FEN between 25 and 74 nM against FEN and between 121 and 900 nM against CAR, indicating higher affinity of ab-FEN against FEN compared to CAR, as one might expect. Considering that FEN concentrations tested were 15-fold higher, these differences might be even higher than they appeared. However, in particular ab-FEN-9 which exhibited an IC_50_-value of 121 ± 23 nM in the receptor activation assay and of 179 ± 21 nM in the binding assay, showed reasonable affinity to CAR. Thus, this antibody could be used as lead molecule for future antibody development and refinement in order to further increase antibody affinity to CAR. As mentioned above, *in vivo* effects of ab-FEN would depend on the binding to the UPSO in the serum, hindering crossing of the BBB by the opioid. Based on our data, we would assume that ab-FEN-9 could bind a sufficient amount of CAR, so that even fatal CAR serum concentrations would not reach the CNS. Further improving affinity of ab-FEN-9 by antibody engineering could reach this goal at even lower antibody concentrations.

Hindering crossing of the BBB by the opioid is very useful before intoxication or shortly after. If antibodies should be suited as a therapy of an intoxication already in progress, they would be more effective after crossing the BBB themselves. Of note, antibody engineering can create protein modifications that allows BBB crossing of antibodies (Zhao et al. 2022a). In such a scenario, the antibody will face the opioid in the presence of its receptor or after the ligand-receptor complex has already been formed. The presence of the receptor might change the interactions between the opioid and the antibody fundamentally. Thus, it is of prime importance to analyze the pharmacodynamics of ab-FEN on the interactions between opioids and their receptor. Our study revealed the first view on the interactions between antibodies, opioids and the µOR expressed in cells. Firstly, we show that ab-FEN are able to break up half of already formed FEN-µOR complexes within ~ 30 min, suggesting that ab-FEN could stop and even reverse already occurring FEN intoxication. Moreover, within 60 min ab-FEN could also break up half of the preformed CAR-µOR complexes; even in the presence of fatal CAR concentrations (10 nM). This time might be sufficient to ensure survival of an intoxicated individual until they obtain medical intensive care. Secondly, our data reveal very high cooperativity of ab-FEN and opioid-activity enhancing ab-FEN effects at low concentrations. Interestingly, FEN enhancing antibody actions were detectable at ab-FEN levels below 15 nM, which corresponds to the FEN concentration used. Thus, at a molecular ratio of FEN/ab-FEN ratio > 1, ab-FEN increased efficacy of FEN. When ab-FEN levels increased over the FEN concentration, the antibody started to inhibit the opioid very efficiently. Of note, this was not observed for ab-FEN-2, indicating that antibody-induced increases in opioid efficacy is an intrinsic property of the antibody, maybe depending on the epitope recognized by the ab-FEN. Opioid enhancing effects of ab-FEN were not observed, when the FEN-µOR complex was already preformed. Based on this notion together with the observation that ab-FEN did not block REMI, we propose a model in which ab-FEN bind the phenethyl group of FEN which is less important for FENs interactions with the receptor (Vo et al. 2021). At FEN/ab-FEN ratios > 1, one antibody molecule binds not more than one FEN molecule and thereby increases its affinity, possibly by increasing locally its concentration around the membrane integrated receptor, when the FC part of the antibody binds to the plasma membrane. This mechanism might be irrelevant when ab-FEN are applied in advance, bind FEN in the serum and thus hinder their crossing of the BBB in the absence of the receptor. However, it might be of dramatic consequences when BBB-crossing antibody fragments would bind to FEN in the presence of the µOR during intoxication. On the other hand, opioid efficacy enhancing effects point to a new clinical application of such antibodies as enhancer or supporter of an opioid-based pain therapy. However, at FEN/ab-FEN ratios < 1, more than one ab-FEN molecule interacts with a single FEN molecule, leading now to the neutralization of the opioid by blocking its binding to the receptor. Such a model would explain both positive cooperativity and bi-functional effects of ab-FEN on FEN. However, further studies are required to confirm this hypothesis. Overall, analysis of the pharmacodynamics of several ab-FEN and various opioids in the presence of the receptor, revealed new insights into the interactions between antibody, opioid and receptor, that will be by useful for future antibody development and refinement, or to determine their proper dosages.

## Conclusion

Our study aimed at establishing cell-based assays to analyze effects of ab-FEN on the interactions of various opioids and the µOR. We discover new antibodies that neutralize lethal CAR concentrations even under conditions of preformed CAR-µOR complexes. We further reveal so far unappreciated effects of ab-FEN on the interactions between FEN and µOR, such as positive cooperativity and opioid activity enhancing effects at low antibody concentrations. Our data help to understand the interactions between antibodies, opioids and receptors on the molecular level better, and thus, will be useful for the future development of an antibody-based therapy against UPSO intoxication.

## Data Availability

Data are availbale upon request. Please contact the corresponding author.

## Abbreviations

cAMP: Cyclic adenosine monophosphate
CAR: Carfentanil
ENDO: Endomorphine-1
FEN: Fentanyl
FSK: Forskolin
IBMX: 3-Isobutyl-1-methylxanthin
MOR: Morphine
µOR: µ Opioid receptors
REMI: Remifentanil
